# The Optimal pressure reactivity index range is disease-specific: A comparison between aneurysmal subarachnoid hemorrhage and traumatic brain injury

**DOI:** 10.1007/s10877-024-01168-9

**Published:** 2024-05-04

**Authors:** Teodor Svedung Wettervik, Timothy Howells, Anders Hånell, Anders Lewén, Per Enblad

**Affiliations:** Department of Medical Sciences, Section of Neurosurgery, Uppsala University, 751 85 Uppsala, Sweden

**Keywords:** Neurointensive care, Outcome, Pressure reactivity index, Subarachnoid hemorrhage, Traumatic brain injury

## Abstract

**Purpose:**

Impaired cerebral pressure autoregulation is common and detrimental after acute brain injuries. Based on the prevalence of delayed cerebral ischemia in aneurysmal subarachnoid hemorrhage (aSAH) patients compared to traumatic brain injury (TBI), we hypothesized that the type of autoregulatory disturbance and the optimal PRx range may differ between these two conditions. The aim of this study was to determine the optimal PRx ranges in relation to functional outcome following aSAH and TBI, respectively.

**Methods:**

In this observational study, 487 aSAH patients and 413 TBI patients, treated in the neurointensive care, Uppsala, Sweden, between 2008 and 2018, were included. The percentage of good monitoring time (%GMT) of PRx was calculated within 8 intervals covering the range from -1.0 to + 1.0, and analyzed in relation to favorable outcome (GOS-E 5 to 8).

**Results:**

In multiple logistic regressions, a higher %GMTs of PRx in the intervals -1.0 to -0.5 and + 0.75 to + 1.0 were independently associated with a lower rate of favorable outcome in the aSAH cohort. In a similar analysis in the TBI cohort, only positive PRx in the interval + 0.75 to + 1.0 was independently associated with a lower rate of favorable outcome.

**Conclusion:**

Extreme PRx values in both directions were unfavorable in aSAH, possibly as high PRx could indicate proximal vasospasm with exhausted distal vasodilatory reserve, while very negative PRx could reflect myogenic hyperreactivity with suppressed cerebral blood flow. Only elevated PRx was unfavorable in TBI, possibly as pressure passive vessels may be a more predominant pathomechanism in this disease.

**Supplementary Information:**

The online version contains supplementary material available at 10.1007/s10877-024-01168-9.

## Introduction

Cerebral blood flow (CBF) disturbances including ischemia and hyperemia are common following acute brain injuries such as aneurysmal subarachnoid hemorrhage (aSAH) and traumatic brain injury (TBI) [23]. Traditionally, cerebral perfusion pressure (CPP) has been used as a surrogate measure of CBF in the neurointensive care (NIC) unit [5, 10]. There are problems with this approach. First, under normal conditions the cerebral autoregulatory capacity maintains CBF stable over a wide range of CPPs. However, following acute brain injury, autoregulation may be impaired and the effects of this on CBF can be difficult to predict [22, 23]. Thus, there has been a great interest in developing methods for continuous monitoring of cerebral pressure autoregulation [20]. The pressure reactivity index (PRx), which is the moving 5-min correlation coefficient between 10 s-values of mean arterial blood pressure (MAP) and intracranial pressure (ICP), is currently the most established estimate of cerebral pressure autoregulation in the NIC [7, 20, 24, 35]. PRx has mostly been studied in TBI, in which higher values, indicative of disrupted cerebral pressure autoregulation, are associated with greater evolution of contusion edema [18], worse cerebral energy metabolism [26, 31], and higher rate of unfavorable functional outcome [7, 24, 36]. PRx intervals have also been used to define a safe and unsafe CPP range, since the combination of high PRx together with low or high CPP has been particularly correlated with worse outcome in TBI, possibly reflecting detrimental ischemia and hyperemia [28]. However, the role of PRx in aSAH is less established. Preliminary studies have found high PRx to be associated with lower CBF [14], a higher risk of developing delayed ischemic neurological deficits (DIND), and a lower rate of favorable outcome [11, 25]. Yet, others have contradicted these results and found no correlation between PRx and DIND [15, 25] or outcome [3, 9, 29]. One reason could be that the predominant mechanisms for CBF disturbances differ between aSAH and TBI, which could have an effect on the physiological and prognostic meaning of certain PRx intervals. For example, high PRx in combination with low CPP is anticipated to indicate proximal vasospasm with exhausted distal vasodilatory reserve resulting in ischemia [23]. At the same time, aSAH patients may develop vasospastic, myogenic hyperreactivity that suppresses CBF [16], but also renders PRx negative. Thus, particularly in aSAH, there may be an autoregulatory “sweet spot” with PRx close to zero, while extreme values in both directions are detrimental. These dual phenomena may differ from TBI, in which pressure passive vessels with either ischemia or hyperemia-induced intracranial hypertension related to high PRx may be more common [17].

To further explore PRx in different acute brain injuries, we aimed to investigate the association between spending a higher percentage of monitoring time within certain PRx intervals in relation to functional outcome following aSAH and TBI. We expected that there would be a roughly linear correlation between PRx and functional outcome in TBI with the quality of patient outcome decreasing across the entire range from -1 to 1. In the case of aSAH patients we hypothesized that there could be a different pattern with regards to PRx due to the known differences in problems relating to cerebral blood flow in these two diseases.

## Materials and Methods

### Patients

In this observational study, we included aSAH or TBI patients, aged 16 and older, with at least 24 h of ICP/PRx data the first 10 days after ictus/injury, and with available long-term functional outcome data from our center between 2008–2018. Of 956 aSAH patients, 420 did not receive an ICP monitor, 42 had less than 24 h of PRx data the first 10 days, and 7 had no outcome data. Thus, the final aSAH cohort included 487 patients. Of 840 TBI patients, 326 did not receive an ICP monitor, 59 had less than 24 h of PRx data the first 10 days, and 42 had no long-term outcome data. Thus, the final TBI cohort included 413 patients.

### Management

The NIC management in TBI [10, 27] and aSAH [4, 19] has been described in detail in previous studies and did not change throughout the study period. In brief, in both diseases, the treatment targets were ICP ≤ 20 mmHg, CPP ≥ 60 mmHg, systolic blood pressure ≥ 100 mmHg, pO_2_ ≥ 12 kPa, arterial glucose 5–10 mM, hemoglobin ≥ 10 g/dL, electrolytes within normal ranges, normovolemia, and body temperature < 38 °C.

In both diseases, unconscious patients (Glasgow Coma Scale Motor [GCS M] score < 6) were intubated, mechanically ventilated, and sedated. Unconscious patients also received ICP monitoring, preferentially an external ventricular drainage (EVD; HanniSet, Xtrans, Smith Medical GmbH, Glasbrunn, Germany) or otherwise an intraparenchymal probe (Codman ICP Micro-Sensor, Codman & Shurtleff, Raynham, MA). Almost all aSAH patients received an EVD as the gold standard for ICP monitoring and to enable drainage of cerebrospinal fluid, while TBI patients with smaller ventricles often received an intraparenchymal monitor. Repeated wake-up tests were performed every day, but the patients were kept sedated in case of intracranial hypertension. Intracranial hematomas with significant mass effect were surgically evacuated. Thiopental and decompressive craniectomy (DC) were last-tier treatments to alleviate refractory increases in ICP [4, 32]. In aSAH, intracranial aneurysms were occluded early by means of endovascular embolization or surgical clipping. Nimodipine was given to all aSAH patients for three weeks post-ictus.

### Outcome

Clinical outcome was assessed by specially trained personnel using structured telephone interviews at 6 (TBI) or 12 (aSAH) months after the brain injury, using the Extended Glasgow Outcome Scale (GOS-E). The scale ranges from 1 (death) to 8 (upper good recovery) [30, 33]. Outcome was dichotomized into favorable and unfavorable corresponding to GOS-E 5–8 and 1–4.

### Cerebral physiological data – acquisition and analysis

The physiological variables (arterial blood pressure (ABP) and ICP) were prospectively collected at high frequency (100 Hz) using the Odin software [12]. The physiological data were automatically stored into Odin via RS232 connections or the local IT-system. Artefacts were removed automatically and manually. ABP was monitored with a radial arterial line at heart level. ICP was monitored with an EVD or an intraparenchymal probe. PRx was calculated as the moving 5-min correlation coefficient of 10 s-averages of ABP and ICP [8, 24]. The percentage of good monitoring time (%GMT) was calculated for PRx in eight separate 0.25-intervals (from -1.00 to + 1.00) for the first 10 days after the aSAH/TBI. The median ICP, CPP, and PRx were also calculated during the first 10 days after ictus/injury.

### Visualizations of PRx insults

The median PRx values were plotted against outcome (GOS-E) in aSAH and TBI, respectively, and both a linear and quadratic fit line were created to explore the nature of the association between these two variables. The effect of PRx on GOS-E for the first 10 days after ictus/injury was also visualized using outcome heatmaps, created in a novel R script, based on two different approaches. In the first approach PRx was divided into 20 segments (hereafter referred to as grid cells) ranging from -1 to + 1, each 0.1 units wide. Then, for each patient, the number of recorded values within each grid cell was divided by the total number of recorded values during the first 10 days for that patient, giving the %GMT for each grid cell. The next step was to calculate a Spearman correlation between %GMT and GOS-E for all patients within each grid cell. A positive correlation indicated that a substantial amount of time with PRx in the range of the grid cell was associated with good outcome, while a negative correlation implied the opposite. It was found when using this strategy that the correlations for grid cells with sparse data tended to approach zero, which would be interpreted as a moderate outcome, neither very good or bad. In this application these “moderate” estimates as a result of sparse data contradicted clinical experience, especially with extreme, and therefore unusual, values known to be dangerous. To mitigate this problem, the data within each grid cell was dichotomized with respect to both GOS-E and %GMT before calculating the Spearman correlation. Since the choice of dichotomization point was not obvious, all possible values were evaluated, as long as they resulted in at least 5 patients on each side of the split. The strongest correlation (highest absolute value) for each grid cell was used, a strategy hereafter referred to as optimized dichotomy. This resulted in a single correlation value for each grid cell, which then was mapped to the jet color scale so that favorable regions were colored blue and detrimental regions colored red. The color scale was limited to correlations within ± 0.50 and results from grid cells with less than 5 patients that had at least 5 min of monitoring time were considered unreliable and therefore colored white.

The second approach aimed to explore whether the association between PRx and outcome changed during the treatment period. To do this the 10-day monitoring period was divided into 30, 8 h long, time intervals which combined with the 20 PRx intervals resulted in 600 grid cells. For each patient the %GMT was calculated for each grid cell by dividing the number of observations within the grid cell by the total number of observations for all grid cells within that time interval. Once all the patients’ %GMTs were calculated the process continued by using optimized dichotomy as described above.

The outcome heatmaps described above were complemented by density graphs to visualize the distribution of observed PRx values. These were constructed by counting the number of observations within each grid cell, and then dividing by the highest count among all grid cells, for each patient. The resulting values were then averaged over all patients and color coded using the jet color scale. To reduce high frequency noise, each pixel was divided into 3 by 3 smaller pixels followed by a Gaussian smoothing (standard deviation = 2).

### Statistical analysis

The statistical analyses were conducted in RStudio software (version 2022.12.0) [1]. Categorical and ordinal/continuous variables were reported as numbers (proportions) and medians (interquartile range [IQR]), respectively. Differences in demography, admission variables, treatments, outcome, and cerebral physiology between aSAH and TBI patients were assessed with the Mann–Whitney or Chi-Square tests. The potential explanatory variables for differences in the %GMT for certain PRx-intervals were assessed using the Spearman correlation test or the Mann–Whitney U-test in the aSAH and TBI cohorts, separately. Furthermore, the %GMT for each PRx interval was correlated with GOS-E using the Spearman correlation test for aSAH and TBI, separately. Similarly, the %GMT for each PRx interval was also used as an independent variable, together with age, GCS M, thiopental, DC, ICP, and CPP, in separate multiple logistic regressions with favorable outcome as the dependent variable. The baseline variables were chosen to account for important prognostic variables of demography (age), injury severity (GCS M), last-tier treatments (thiopental and DC), and the main NIC treatment targets (ICP and CPP). A *p*-value below 0.05 was considered statistically significant.

### Ethics

The study was approved by the Swedish Ethical Review Authority. Written, informed consent was obtained in most cases, but was waived if the patients and their relatives could no longer be reached.

## Results

### Demography, admission variables, treatments, and clinical outcome

The 487 aSAH patients were slightly older than the 413 TBI patients (*p* < 0.001) and more often females than males, while the TBI cohort was predominately males (*p* < 0.001; see Table 1 for details). At admission, the aSAH patients exhibited a higher GCS M score (*p* < 0.001) and a lower rate of unreactive pupil(s). There was no difference in the treatment rate of thiopental or DC between the two cohorts (*p* > 0.05). At follow-up, the aSAH patients showed a higher mortality (*p* = 0.01) and a lower rate of favorable outcome (*p* < 0.001).

**Table 1:** Demography, admission variables, treatments, and functional outcome in the aSAH and TBI cohort

	aSAH	TBI	*p-*value
Patients, *n* (%)	487 (54%)	413 (46%)	NA
Age, median (IQR)	59 (51–67)	52 (31–65)	< *0.001*
Sex (male/female), *n* (%)	160/327 (33/67%)	324/89 (78/22%)	< *0.001*
GCS M at admission, median (IQR)	6 (5–6)	5 (4–5)	< *0.001*
Pupillary status (unreactive), *n* (%)	28 (6%)	79 (19%)	< *0.001*
ICP-monitoring (intraparenchymal/EVD/both), *n* (%)	9/444/34 (2/91/7%)	237/80/96 (57/19/23%)	< *0.001*
Thiopental, *n* (%)	51 (10%)	52 (13%)	0.32
DC, *n* (%)	51 (10%)	45 (11%)	0.84
GOS-E, median (IQR)	3 (3–5)	5 (3–7)	< *0.001*
Favorable outcome, *n* (%)	151 (31%)	221 (54%)	< *0.001*
Mortality, *n* (%)	111 (23%)	66 (16%)	*0.01*

Italics indicate statistical significance

aSAH = Aneurysmal subarachnoid hemorrhage. DC = Decompressive craniectomy. EVD = External ventricular drainage. GCS M = Glasgow Coma Scale Motor score. GOS-E = Glasgow Outcome Scale-Extended. ICP = Intracranial pressure. IQR = Interquartile range. TBI = Traumatic brain injury

### Cerebral physiology during the first 10 days after the acute brain injury

In the aSAH and TBI cohorts (Supplementary Table 1), respectively, the median ICP first 10 days after brain injury was 12 (IQR 10–14) and 11 (IQR 8–15) mmHg, the median CPP was 79 (IQR 75–86) and 76 (IQR 71–82) mmHg, and the median PRx was + 0.21 (IQR + 0.10- + 0.32) and + 0.05 (IQR -0.05- + 0.15). The aSAH patients spent less %GMT with a PRx within the interval -1.0 to 0.0, and a higher %GMT with a PRx within the interval + 0.25 to + 1.0, than the TBI patients (*p* < 0.001).

### %GMT in certain PRx intervals in relation to demography, admission variables, and treatments

In the aSAH cohort, the association between PRx and age, GCS M, thiopental/DC treatment, ICP, and CPP are presented in Table 2 and Supplementary Table 2. In brief, younger age, higher GCS M, no thiopental/DC, lower ICP, and higher CPP were more common when PRx was closer to zero than extreme values in both directions.

**Table 2:** The percentage of monitoring time within certain PRx intervals vs. demography, admission variables, and cerebral physiology

PRx interval	aSAH				TBI			
	Age	GCS M	ICP	CPP	Age	GCS M	ICP	CPP
-1.0 ≤ PRx ≤ -0.75 (%GMT)	-0.03	*-0.20*^*c*^	0.08	-0.07	*-0.26*^*c*^	0.04	*0.10*^*a*^	*-0.19*^*c*^
-0.75 < PRx ≤ -0.50 (%GMT)	-0.03	*-0.14*^*b*^	0.01	0.02	*-0.26*^*c*^	0.04	-0.02	*-0.11*^*a*^
-0.50 < PRx ≤ -0.25 (%GMT)	-0.08	*-0.10*^*a*^	-0.06	0.08	*-0.31*^*c*^	0.06	-0.09	*-0.12*^*a*^
-0.25 < PRx ≤ 0.00 (%GMT)	*-0.13*^*b*^	-0.02	-0.07	*0.11*^*a*^	*-0.22*^*c*^	0.06	*-0.10*^*a*^	-0.06
0.00 < PRx ≤ + 0.25 (%GMT)	*-0.10*^*a*^	*0.19*^*c*^	*-0.16*^*c*^	*0.21*^*c*^	*0.23*^*c*^	*0.10*^*a*^	*-0.11*^*a*^	*0.23*^*c*^
+ 0.25 < PRx ≤ + 0.50 (%GMT)	0.07	*0.29*^*c*^	*-0.25*^*c*^	*0.25*^*c*^	*0.35*^*c*^	0.06	-0.05	*0.28*^*c*^
+ 0.50 < PRx ≤ + 0.75 (%GMT)	*0.13*^*b*^	*0.09*^*a*^	-0.08	0.04	*0.26*^*c*^	-0.05	0.00	*0.14*^*b*^
+ 0.75 < PRx ≤ + 1.0 (%GMT)	*0.16*^*c*^	*-0.19*^*c*^	*0.15*^*b*^	*-0.37*^*c*^	-0.08	*-0.20*^*c*^	0.07	*-0.22*^*c*^

The table indicates the Spearman correlation coefficient between %GMT of each PRx interval vs. age, GCS M, ICP, and CPP, for aSAH and TBI patients, respectively. ^a^*p* < 0.05. ^b^*p* < 0.01, ^c^*p* < 0.001. Italics indicate statistical significance

aSAH = Aneurysmal subarachnoid hemorrhage. CPP = Cerebral perfusion pressure. GCS M = Glasgow Coma Scale Motor score. GMT = Good monitoring time. GOS-E = Glasgow Outcome Scale-Extended. ICP = Intracranial pressure. PRx = Pressure reactivity index. TBI = Traumatic brain injury

In the TBI cohort, the association between PRx and age, GCS M, thiopental/DC treatment, ICP, and CPP are presented in Table 2. In brief, older age, lower GCS M, and thiopental/DC treatment were usually associated with PRx in the higher interval.

### Linear and quadratic fits for PRx versus GOS-E

**Fig. 1:**
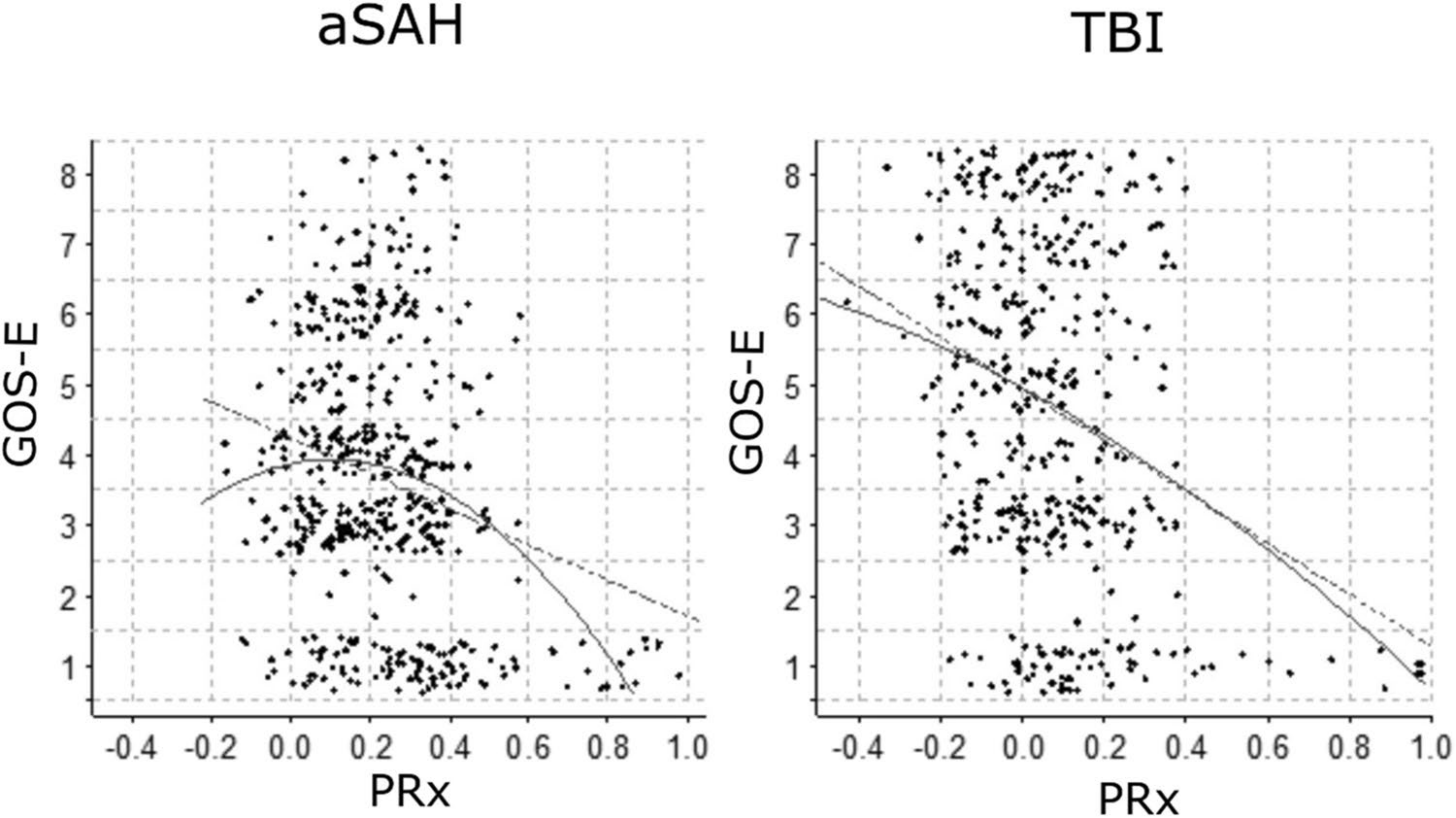
Linear and quadratic fit of PRx vs. GOS-E in aSAH and TBI. The figure shows the scatter plot for median PRx versus the GOS-E patient outcome over the first 10 days following injury for the aSAH cohort (Left) and for the TBI cohort (Right), with the linear fits (dotted lines) and the quadratic fits (solid lines). To avoid over-plotting in the scatter plots and make the density variations more visible within each GOS-E category, a random jitter of -0.4 to 0.4 was added to GOS-E. aSAH = Aneurysmal subarachnoid hemorrhage. GOS-E = Glasgow Outcome Scale-Extended. PRx = Pressure reactivity index. TBI = Traumatic brain injury

Figure 1 shows a scatter plot for the TBI cohort with median PRx on the x-axis and GOS-E on the y-axis together with the linear (*p* < 0.01 for the linear coefficient) and quadratic (*p* = 0.4 for the quadratic coefficient) fits to the data. The two fits appear similar, with both showing an optimal PRx for more negative values. Figure 1 also shows the same data for the aSAH cohort. In this case the two fits are very different, with the linear fit (*p* < 0.01 for the linear coefficient) showing an optimal PRx of minus one, while the quadratic fit (*p* < 0.01 for the quadratic coefficient) shows an optimal PRx of + 0.10.

### %GMT in certain PRx intervals in relation to outcome

**Fig. 2:**
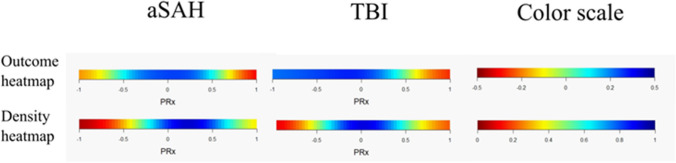
PRx distribution and correlation with outcome in aSAH and TBI. Outcome heatmap—The outcome heatmap for the aSAH and TBI patients indicate the color-coded correlation coefficient between %GMT of PRx for that interval vs. GOS-E. Red color indicates an association between a higher %GMT and lower GOS-E (worse outcome), whereas blue color indicates the opposite association. As indicated by the figures, aSAH patients benefitted from a PRx between -0.50 to + 0.50, while extreme values in both directions were unfavorable. However, TBI patients overall benefitted from lower PRx values. Density heatmap—The density heatmaps indicate the data frequency of PRx values. Blue color indicates highly frequent PRx values, while red color indicates that they were rare. As indicated, PRx between -0.50 to + 0.50 was most frequent in both the aSAH and TBI cohort. aSAH = Aneurysmal subarachnoid hemorrhage. GMT = Good monitoring time. PRx = Pressure reactivity index. TBI = Traumatic brain injury

**Fig. 3:**
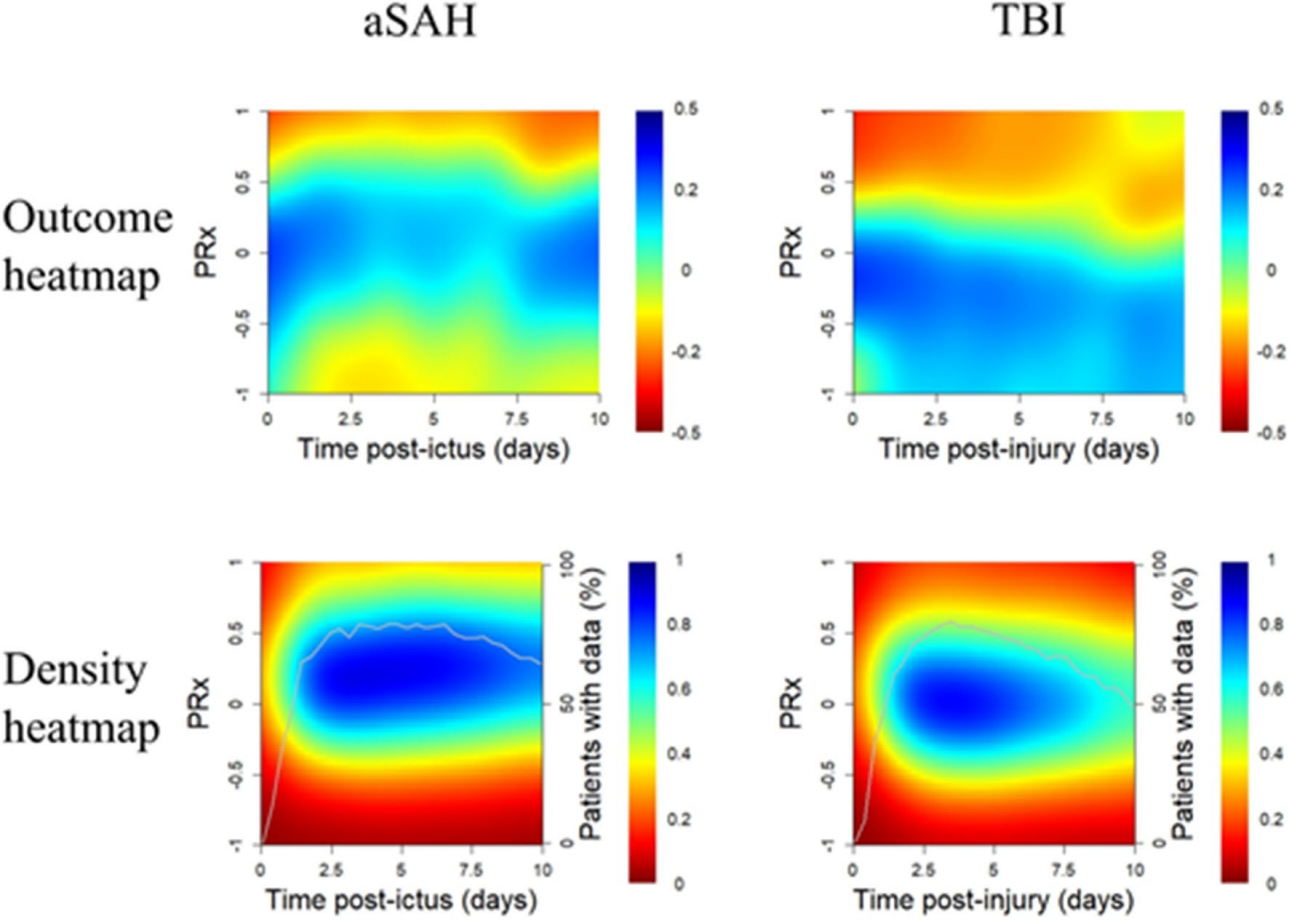
PRx distribution and correlation with outcome in aSAH and TBI during the first 10 days post-ictus/injury. Outcome heatmap—The outcome heatmap for the aSAH and TBI patients indicate the color-coded correlation coefficient between %GMT of PRx for that interval vs. GOS-E in relation to time after ictus/injury. Red color indicates an association between a higher %GMT and lower GOS-E (worse outcome), whereas blue color indicates the opposite association. As indicated by the figures, aSAH patients favored from a PRx between -0.50 to + 0.50, while extreme values in both directions were unfavorable. However, TBI patients overall benefitted from lower PRx values. The optimal PRx intervals appeared fairly stable over the first 10 days. Density heatmap—The density heatmaps indicate the data frequency of PRx values at certain time points after ictus/injury. Blue color indicates highly frequent PRx values, while red color indicates that they were rare. As indicated, PRx between -0.50 to + 0.50 was most frequent in both the aSAH and TBI cohort. The white line indicates the proportion of patients for the entire aSAH/TBI cohort that contributed data for that time window. aSAH = Aneurysmal subarachnoid hemorrhage. GMT = Good monitoring time. PRx = Pressure reactivity index. TBI = Traumatic brain injury

In univariate analyses of the correlation between %GMT for certain PRx intervals and GOS-E in the aSAH cohort (Table 3 and Fig. 2), a higher %GMT of PRx in the intervals -0.50 to + 0.50 correlated with higher GOS-E, whereas the opposite was found for the intervals -1.00 to -0.75 and + 0.75 to + 1.00. These associations appeared consistent throughout the 10-day course (Fig. 3). In multiple logistic regressions of favorable outcome in which each PRx interval was evaluated separately (Table 4), a higher %GMT in the intervals 0.00 to + 0.50 was independently associated with a higher rate of favorable outcome, while the opposite was found for the intervals -1.00 to -0.50 and + 0.75 to + 1.00. Age, GCS M, thiopental, DC, ICP, and CPP were also included as baseline variables in these regressions.

**Table 3:** The percentage of monitoring time within certain PRx intervals vs. GOS-E in aSAH and TBI – a Spearman rank correlation analysis

	aSAH	TBI
-1.0 ≤ PRx ≤ -0.75 (%GMT)	*-0.17*^*c*^	*0.13*^*b*^
-0.75 < PRx ≤ -0.50 (%GMT)	-0.03	*0.18*^*c*^
-0.50 < PRx ≤ -0.25 (%GMT)	*0.11*^*a*^	*0.22*^*c*^
-0.25 < PRx ≤ 0.00 (%GMT)	*0.20*^*c*^	*0.21*^*c*^
0.00 < PRx ≤ + 0.25 (%GMT)	*0.35*^*c*^	0.00
+ 0.25 < PRx ≤ + 0.50 (%GMT)	*0.24*^*c*^	*-0.11*^*a*^
+ 0.50 < PRx ≤ + 0.75 (%GMT)	-0.07	*-0.18*^*c*^
+ 0.75 < PRx ≤ + 1.0 (%GMT)	*-0.37*^*c*^	*-0.16*^*b*^

The table indicates the Spearman correlation coefficient between %GMT of each PRx interval vs. GOS-E for aSAH and TBI. ^a^*p* < 0.05. ^b^*p* < 0.01, ^c^*p* < 0.001. Italics indicate statistical significance

aSAH = Aneurysmal subarachnoid hemorrhage. GMT = Good monitoring time. GOS-E = Glasgow Outcome Scale-Extended. PRx = Pressure reactivity index. TBI = Traumatic brain injury

**Table 4:** Multiple logistic outcome regressions of favorable outcome in aSAH and TBI

PRx interval [regression number]	aSAH (a)		TBI (b)	
	OR (95% CI)	*p*-value	OR (95% CI)	*p*-value
-1 ≤ PRx ≤ -0.75 (GMT) [1]	0.57 (0.35–0.88)	*0.02*	1.05 (0.86–1.29)	0.64
-0.75 < PRx ≤ -0.50 (GMT) [2]	0.92 (0.84–0.996)	*0.046*	1.02 (0.97–1.08)	0.48
-0.50 < PRx ≤ -0.25 (GMT) [3]	0.99 (0.94–1.03)	0.56	1.03 (0.99–1.06)	0.24
-0.25 < PRx ≤ 0.00 (GMT) [4]	1.00 (0.96–1.03)	0.78	1.02 (0.99–1.07)	0.21
0.00 < PRx ≤ + 0.25 (GMT) [5]	1.09 (1.03–1.14)	*0.002*	1.01 (0.97–1.06)	0.48
+ 0.25 < PRx ≤ + 0.50 (GMT) [6]	1.05 (1.01–1.09)	*0.02*	1.00 (0.97–1.04)	0.84
+ 0.50 < PRx ≤ + 0.75 (GMT) [7]	0.99 (0.96–1.02)	0.65	0.98 (0.94–1.02)	0.38
+ 0.75 < PRx ≤ + 1.0 (GMT) [8]	0.93 (0.88–0.98)	*0.02*	0.91 (0.84–0.97)	*0.01*

In addition to the PRx-variable, the regressions included age, GCS M, thiopental, DC, median ICP (10 days), and CPP (10 days) as baseline variables

Regression statistics:

1a; AUROC (95%CI) = 0.80 (0.75–0.84), AIC = 494, R^2^ = 0.33

1b; AUROC (95%CI) = 0.77 (0.73–0.82), AIC = 494, R^2^ = 0.27

2a; AUROC (95%CI) = 0.79 (0.74–0.84), AIC = 494, R^2^ = 0.32

2b; AUROC (95%CI) = 0.77 (0.73–0.81), AIC = 494, R^2^ = 0.27

3a; AUROC (95%CI) = 0.79 (0.75–0.83), AIC = 498, R^2^ = 0.31

3b; AUROC (95%CI) = 0.77 (0.73–0.82), AIC = 493, R^2^ = 0.27

4a; AUROC (95%CI) = 0.79 (0.75–0.83), AIC = 498, R^2^ = 0.31

4b; AUROC (95%CI) = 0.77 (0.73–0.82), AIC = 492, R^2^ = 0.27

5a; AUROC (95%CI) = 0.80 (0.76–0.84), AIC = 488, R^2^ = 0.33

5b; AUROC (95%CI) = 0.77 (0.73–0.82), AIC = 494, R^2^ = 0.27

6a; AUROC (95%CI) = 0.79 (0.75–0.84), AIC = 492, R^2^ = 0.32

6b; AUROC (95%CI) = 0.77 (0.73–0.82), AIC = 494, R^2^ = 0.27

7a; AUROC (95%CI) = 0.79 (0.75–0.83), AIC = 498, R^2^ = 0.31

7b; AUROC (95%CI) = 0.77 (0.73–0.82), AIC = 493, R^2^ = 0.27

8a; AUROC (95%CI) = 0.80 (0.76–0.84), AIC = 491, R^2^ = 0.33

8b; AUROC (95%CI) = 0.78 (0.73–0.82), AIC = 483, R^2^ = 0.30

AIC = Akaike information criteria. aSAH = Aneurysmal subarachnoid hemorrhage. AUROC = Area under receiver operating characteristics curve. CI = Confidence interval. CPP = Cerebral perfusion pressure. DC = Decompressive craniectomy. GCS M = Glasgow Coma Scale Motor score. GMT = Good monitoring time. ICP = Intracranial pressure. OR = Odds ratio. PRx = Pressure reactivity index. TBI = Traumatic brain injury

In univariate analyses of the correlation between %GMT for certain PRx intervals and GOS-E in the TBI cohort (Table 3 and Fig. 2), a higher %GMT of PRx in the intervals -1.00 to 0.00 correlated with higher GOS-E, while the opposite was found in the interval + 0.25 to + 1.00. These associations appeared consistent throughout the 10-day course, but the transition towards worse outcome slightly decreased with each day post-injury (Fig. 3). In multiple logistic regressions of favorable outcome in which each PRx interval was evaluated separately (Table 4), a lower %GMT in the intervals 0.75 to + 1.00 was independently associated with a higher rate of favorable outcome. Age, GCS M, thiopental, DC, ICP, and CPP were also included as baseline variables in these regressions.

## Discussion

In this observational study, based on 487 aSAH and 413 TBI patients with high-frequency physiological data, we tested the hypothesis that aSAH and TBI patients exhibit disease-specific optimal PRx intervals. An initial test looked at linear and quadratic fits to scatter plots of 10-day median PRx versus GOS-E in the TBI and aSAH cohorts. In TBI, the two fits were similar, since both indicated an optimal PRx of -1, but in aSAH they were very different, with the quadratic fit indicating an optimal PRx of + 0.10. We wanted a more stringent test of this unexpected result with measures of statistical significance and controlling for confounders. This required a more detailed analysis than the usual approach based on dichotomizing the PRx range into favorable and unfavorable intervals using a single threshold. Instead, we divided the range into 8 bins which were evaluated separately as predictive variables, first in a univariate analysis (Spearman correlation), and then in a multivariate logistic regression.

The differences between the aSAH results and TBI are clear in the univariate analysis (Table 3). The optimal PRx for the aSAH cohort was in the middle range of -0.25 to + 0.25, with significant declines to the extreme positive and negative bins which were both significantly associated with unfavorable outcome. Optimal PRx for the TBI cohort was in the negative range. The only nonsignificant results were in the transitional regions between favorable and unfavorable outcome.

In the multivariate analysis (Table 4), the results for the aSAH cohort were very consistent with the univariate. For the TBI cohort, while the trend was similar to the univariate results, the only statistically significant result was the association of PRx between + 0.75 and + 1.0 with a lower rate of favorable outcome. Therefore, the unexpected quadratic fit for PRx in SAH (Fig. 1) actually held up better when controlling for confounders like age and severity of injury than the familiar linear fit in TBI (Fig. 1), This suggests that pressure reactivity and related cerebrovascular metabolic autoregulation mechanisms, or disturbances of these mechanisms, may play a more important role in recovery or deterioration following SAH than they do following TBI.

Our study supports numerous previous findings that higher PRx is detrimental in TBI [7, 24, 36]. We found that there was a transition from favorable towards unfavorable outcome when PRx exceeded 0.0 to + 0.3, consistent with previous studies [21, 36]. Based on the large body of studies on PRx in TBI [7, 21, 24, 35], the bi-directional unfavorable PRx zones in aSAH were unexpected. At the same time, this finding may reconcile the conflicting results on the role of PRx in aSAH in previous studies [3, 9, 11, 15, 25, 29]. Understandably, these previous studies have assumed that the linear model of PRx used in TBI would hold up in aSAH, and have analyzed mean PRx values and %GMT above and below single PRx threshold values. These analytical approaches are flawed given the quadratic model of PRx we observe in SAH. In this case a mean value in the safe range could misleadingly combine extreme values in both directions outside the safe range. In the case of a %GMT threshold, at least two carefully selected thresholds are needed to identify the safe range for PRx following SAH.

**Fig. 4:**
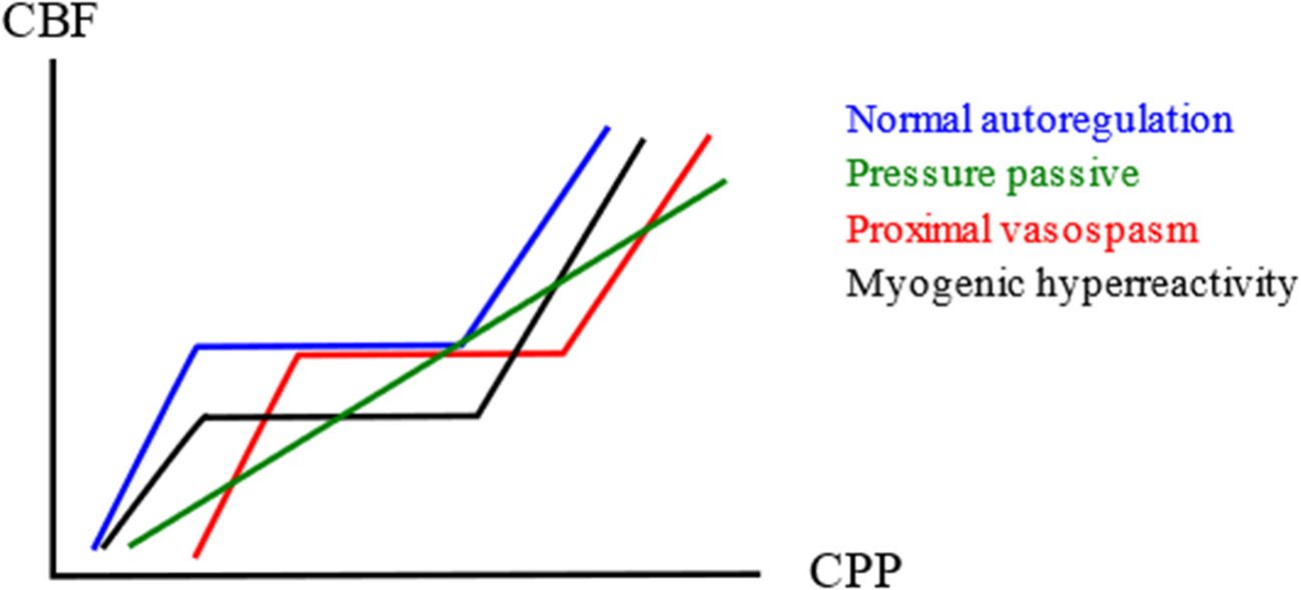
CBF and autoregulatory disturbances in aSAH and TBI. The figure illustrates the “Lassen curve” of autoregulation. Normally, CBF is autoregulated within a large range of CPPs, but this function may become disrupted after acute brain injury. The cerebral vessels could become pressure passive, so that CBF is directly proportional to the CPP, leading to ischemia or hyperemia depending on the CPP level. In cases with proximal vasospasm, the curve may become right shifted so that the distal vasodilatory reserve is exhausted at a higher CPP level. In cases with distal vasospasm, we speculate whether myogenic hyperreactivity could yield both a suppressed CBF and highly reactive vessels. aSAH = Aneurysmal subarachnoid hemorrhage. CBF = Cerebral blood flow. CPP = Cerebral perfusion pressure. TBI = Traumatic brain injury.

This study also supports the view that the differences in the optimal PRx range between the diseases could be linked to the underlying etiology of the autoregulatory disturbances. In TBI, Martin et al. demonstrated that pressure passive vessels with cerebral ischemia followed by hyperemia is the main autoregulatory disturbance in the acute phase after TBI [17]. Pressure passive cerebral vessels are associated with positive PRx values, more secondary brain injury, and a lower rate of favorable outcome. TBI patients also exhibit vasospasm in the late phase to some extent [17], but this mechanism is much more pronounced in aSAH and occurs to different degrees in both the proximal and distal cerebral vessels [6]. It has been suggested that the distribution of vasospasm and autoregulatory disturbances throughout the cerebrovascular tree may result in different types of Lassen curves (Fig. 4) [16]. Patients with more pronounced proximal cerebral vasospasm with intact distal cerebral pressure autoregulation, would be expected to exhibit a right-shifted autoregulatory curve and to display high PRx values near the lower limit of autoregulation. However, in case of distal cerebral vasospasm with myogenic hyperreactivity, the CBF could be suppressed while the vessels are still very reactive and therefore render PRx negative. These hypotheses need to be externally validated in other cohorts and further evaluated in relation to multimodal monitoring data of CBF, brain tissue oxygenation, and energy metabolism. If they hold true, it would be interesting to further explore the ways in which aSAH patients with either very low or high PRx might respond differently to therapies aimed at CBF augmentation. Patients with exhausted distal vasodilatory reserve would be expected to respond better to fluid and possibly inotrope treatments to increase CPP. However, in cases with hyperreactive distal vessels, it is possible that intra-arterial vasodilators would be more effective.

The variables controlled for in the regression analysis were based on demography, admission status, treatments, and cerebral physiology correlated with the %GMT within the eight PRx bins differently in the aSAH and TBI cohorts. The main findings were that worse admission status and last-tier treatments of high ICP correlated both with a higher %GMT of both very low and high PRx in the aSAH cohort, while they mainly correlated with higher %GMT of high PRx in the TBI cohort. These associations appear to reflect the fact that patients with worse injury status also exhibited worse PRx patterns for their type of brain injury. However, it is noteworthy, that a higher %GMT of PRx in very low and high intervals for aSAH and the highest interval for TBI remained statistically significant in the multiple logistic regressions of favorable outcome after adjustment for these potential confounders.

### Methodological considerations

The main strengths of this manuscript were the large cohort sizes of the aSAH and TBI patients with high-frequency data on cerebral physiology, detailed clinical information, and long-term GOS-E.

There were also some limitations. First, our analyses between PRx and outcome were associations, possibly causal to some extent, but may reflect many factors such as demography, underlying brain damage, and the local NIC management protocol. We did address this to some extent by proceeding with multiple logistic outcome regressions to adjust for baseline variables. Second, GOS-E is a complex outcome measure which is related to many factors such as patient age, primary brain injury, secondary insults, treatments, and neurorehabilitation. The aSAH and TBI patients differed in many of these aspects, which might have also affected the relative importance of PRx. In addition, the associations between PRx and GOS-E were often weak, which was expected considering the plethora of variables that influence long-term outcome. Furthermore, it has been questioned if PRx/CPPopt are reliable in case of an open EVD or post-DC. Using an EVD system with a certain outflow resistance preserves much of the ICP amplitude when the EVD is open and makes the measurements sensitive for very rapid ICP changes which is a prerequisite for reliable PRx calculations that were based on 10 s averages of high-resolution data. Several studies support that these measures remain valid in these scenarios [2, 13, 34], and we therefore decided not to exclude these patients. In addition, the median %GMT of PRx in the very negative range was small, which makes these analyses less reliable and possibly less clinically relevant. Still, although it was rare, time spent in this PRx range was strongly correlated with unfavorable outcome in the aSAH cohort, indicating that it contained highly relevant physiological information. Lastly, our visualization methods were based on the principle of optimized dichotomy of outcome for each grid cell. This method was particularly suitable to take into account that extreme values of PRx were unusual, but detrimental. Alternative approaches, for example a fixed dichotomization of GOS-E 1–4 vs. 5–8, would likely have been less sensitive for the detection of this effect.

## Conclusion

aSAH patients exhibited a quadratic association between PRx and outcome, as extreme values in both directions were unfavorable and values close to zero were optimal. This may explain the conflicting findings from previous studies on the physiological and prognostic significance of PRx, since the negative effect of extreme values may be evened out by mean value assessments and the %GMT above threshold may not capture the detrimental role of very negative PRx. We speculate whether high PRx in aSAH could indicate proximal vasospasm with exhausted distal cerebrovascular reserve, while very negative PRx could reflect myogenic hyperreactivity with suppressed CBF. In TBI patients, the association between PRx and outcome was linear and there was a transition towards worse outcome in the interval 0.00 to + 0.30, highly consistent with previous studies.

## Supplementary Information

Below is the link to the electronic supplementary material.

Supplementary file1 (DOCX 17 KB)

## Data Availability

Not available.
