# Supplementary material for: The Optimal pressure reactivity index range is disease-specific: A comparison between aneurysmal subarachnoid hemorrhage and traumatic brain injury
Source: J Clin Monit Comput. 2024 May 4;38(5):1089–99. doi: 10.1007/s10877-024-01168-9 (PMC11427507; doi:10.1007/s10877-024-01168-9)
Supplement: Supplementary file 1 — Supplementary file1 (DOCX 17 KB) [file 10877_2024_1168_MOESM1_ESM.docx]

**Supplementary table 1. Cerebral physiological variables the first 10 days after brain injury.**

| **Variables** | **aSAH** | **TBI** | **p-value** |
| --- | --- | --- | --- |
| ICP (mmHg), median (IQR) | 12 (10-14) | 11 (8-15) | ***0.001*** |
| CPP (mmHg), median (IQR) | 79 (75-86) | 76 (71-82) | ***< 0.001*** |
| PRx (coefficient), median (IQR) | +0.21 (+0.10-+0.32) | +0.05 (-0.05-+0.15) | ***< 0.001*** |
| *PRx intervals* | | | |
| -1 ≤ PRx ≤ -0.75 (%GMT), median (IQR) | 0.3 (0.1-0.7) | 0.9 (0.5-1.5) | ***< 0.001*** |
| -0.75 < PRx ≤ -0.50 (%GMT), median (IQR) | 2.5 (1.4-4.8) | 5.1 (3.2-8.5) | ***< 0.001*** |
| -0.50 < PRx ≤ -0.25 (%GMT), median (IQR) | 8.4 (5.1-12.2) | 14.1 (9.6-19.8) | ***< 0.001*** |
| -0.25 < PRx ≤ 0.00 (%GMT), median (IQR) | 17.2 (13.0-21.2) | 23.1 (18.9-26.1) | ***< 0.001*** |
| 0.00 < PRx ≤ +0.25 (%GMT), median (IQR) | 23.1 (20.0-26.0) | 22.8 (19.5-26.8) | 0.48 |
| +0.25 < PRx ≤ +0.50 (%GMT), median (IQR) | 22.7 (18.1-26.6) | 16.7 (12.2-21.8) | ***< 0.001*** |
| +0.50 < PRx ≤ +0.75 (%GMT), median (IQR) | 14.9 (10.8-20.9) | 8.4 (6.0-12.5) | ***< 0.001*** |
| +0.75 < PRx ≤ +1.0 (%GMT), median (IQR) | 5.0 (2.8-8.5) | 2.8 (1.7-4.5) | ***< 0.001*** |

aSAH = Aneurysmal subarachnoid hemorrhage. CPP = Cerebral perfusion pressure. GMT = Good monitoring time. ICP = Intracranial pressure. IQR = Interquartile range. PRx = Pressure reactivity index. TBI = Traumatic brain injury.

**Supplementary table 2. The percentage of monitoring time within certain PRx intervals vs. treatments**

| **PRx interval** | **aSAH** | | | | **TBI** | | | |
| --- | --- | --- | --- | --- | --- | --- | --- | --- |
|  | Thiopental | | DC | | Thiopental | | DC | |
|  | Yes | No | Yes | No | Yes | No | Yes | No |
| -1 ≤ PRx ≤ -0.75 (%GMT) | ***0.5 (0.2-0.9)^a^*** | ***0.3 (0.1-0.6)^a^*** | ***0.5 (0.3-0.7)^b^*** | ***0.3 (0.1-0.6)^b^*** | ***1.3 (0.8-2.5)^c^*** | ***0.8 (0.4-1.4)^c^*** | 0.9 (0.5-1.3) | 0.8 (0.5-1.5) |
| -0.75 < PRx ≤ -0.50 (%GMT) | 2.1 (1.5-4.2) | 2.5 (1.4-4.8) | 2.8 (1.7-5.8) | 2.4 (1.4-4.7) | 4.7 (3.2-5.9) | 5.3 (3.2-8.7) | 4.5 (3.1-6.9) | 5.3 (3.2-8.6) |
| -0.50 < PRx ≤ -0.25 (%GMT) | ***6.1 (4.5-10.5)^a^*** | ***8.5 (5.2-12.2)^a^*** | 8.9 (5.4-13.1) | 8.3 (5.0-12.1) | ***11.6 (7.6-16.4)^b^*** | ***14.4 (10.0-20.4)^b^*** | ***11.8 (7.8-16.7)^a^*** | ***14.5 (10.0-20.3)^a^*** |
| -0.25 < PRx ≤ 0.00 (%GMT) | ***14.8 (10.7-17.4)^c^*** | ***17.6 (13.4-21.4)^c^*** | 16.7 (12.5-20.8) | 17.3 (13.2-21.2) | ***20.8 (15.6-24.8)^b^*** | ***23.5 (19.4-26.2)^b^*** | ***20.4 (16.9-25.1)^a^*** | ***23.3 (19.3-26.2)^a^*** |
| 0.00 < PRx ≤ +0.25 (%GMT) | ***20.0 (15.3-23.6)^c^*** | ***23.4 (20.4-26.3)^c^*** | 22.5 (18.9-24.5) | 23.3 (20.0-26.1) | 22.2 (19.3-26.0) | 23.1 (19.5-26.9) | 22.6 (19.5-25.8) | 23.0 (19.5-26.8) |
| +0.25 < PRx ≤ +0.50 (%GMT) | 19.3 (15.6-23.7)^c^ | ***23.1 (18.5-26.8)^c^*** | 22.2 (18.4-23.7) | 22.8 (18.1-26.8) | 17.7 (14.2-21.1) | 16.6 (11.9-22.0) | 17.1 (14.0-20.3) | 16.6 (11.9-22.1) |
| +0.50 < PRx ≤ +0.75 (%GMT) | ***19.1 (11.5-21.9)*** | ***14.8 (10.7-20.4)*** | 15.3 (11.0-20.8) | 14.9 (10.7-21.0) | 9.2 (6.5-14.4) | 8.3 (5.9-12.3) | ***11.0 (7.3-14.7)^a^*** | ***8.2 (5.8-12.2)^a^*** |
| +0.75 < PRx ≤ +1.0 (%GMT) | ***10.8 (5.9-23.3)^c^*** | ***4.6 (2.7-7.7)^c^*** | 6.0 (3.1-10.2) | 4.9 (2.8-8.3) | ***4.2 (2.1-8.6)^c^*** | ***2.7 (1.6-4.2)^c^*** | ***4.2 (2.3-7.0)^c^*** | ***2.6 (1.6-4.3)^c^*** |

The numbers indicate the median (IQR) for each subgroup of patients.

^a^p < 0.05. ^b^p<0.01, ^c^p<0.001. Bold and italics indicate statistical significance.

aSAH = Aneurysmal subarachnoid hemorrhage. DC = Decompressive craniectomy. GMT = Good monitoring time. IQR = Interquartile range. PRx = Pressure reactivity index. TBI = Traumatic brain injury.
